# Shorter Hours for Welsh Mothers

**Published:** 1920-09-11

**Authors:** 


### Shorter Hours for Welsh Mothers.

A stir has been caused in South Wales by the annual report of a district medical officer of health Dr. T. Baillie Smith, who as guardian of the public health of the mining district of Abertillery, has become the hero of the colliers' wives. Shorter hours and more rest for mothers is the gospel he preaches in his report. "All of us know," he says, "how the workers of the country are agitating for shorter hours, but how is the mother of a large family to obtain shorter hours and more rest?"

His suggestions for lightening mothers' work may be tabulated thus:—Pit-head baths, for which the miners themselves have been agitating for several years past, the employment of a tailor at the pit to do rough repairs to working clothes, the keeping of pit clothes entirely out of the house, as is the practice in Germany, where the miner changes his working apparel before leaving the colliery, the provision of household helps at a moderate cost to mothers who have families and are confined in their own homes, such helps to take charge of the house, do all the buying, cooking, and cleaning, and attend to the children. The suggestion as to helps for working mothers has brought forth a chorus of approval; and all social observers are agreed that the miner's wife, working a seventeen-hour day against her husband's seven-hour day, as Mr. Edgar Chappell, a Ministry of Health inspector puts it, needs more time off; but so expert an authority as Mrs. Watts Morgan, wife of the miners' M.P., puts her veto on the tailor proposal, remarking that the wife of the miner would not consent to forgo the privilege of repairing her husband's clothes.

